# Cross-cultural study of religiosity, gratitude, and happiness: self-esteem as a mediator among Malaysian and Indonesian adults

**DOI:** 10.1186/s40359-026-04032-4

**Published:** 2026-01-28

**Authors:** Azmawaty Mohamad Nor, Nursyuhaidah Mohd Kadri, Rozita Wahab, Lusy Asa Akhrani, Ika Rahma Susilawati

**Affiliations:** 1 Department of Educational Psychology and Counselling, Faculty of Education, Universiti Malaya, Kuala Lumpur, Malaysia; 2 Department of Psychology, Faculty of Social and Political Science, Universitas Brawijaya, Malang, Indonesia

**Keywords:** Happiness, Religiosity, Gratitude, Self-esteem, Cross-cultural, Adults

## Abstract

**Background:**

Happiness is a central goal of human life and a core focus of positive psychology, yet its predictors and pathways often vary across different cultural contexts. Religiosity, gratitude, and self-esteem have each been linked to happiness. However, little is known about how these constructs interact in collectivist societies, such as Malaysia and Indonesia. This study examines cross-cultural differences in happiness by investigating how religiosity and gratitude predict happiness, with self-esteem as a mediator, among Malaysian and Indonesian adults.

**Methods:**

A total of 739 adults (40.6% Malaysian and 59.4% Indonesian; age 18–72 years) were selected using a voluntary sampling method. Data were collected using a set of questionnaire-based surveys through a cross-sectional study and then analysed using structural equation modelling (SEM).

**Results:**

Religiosity, gratitude, and self-esteem significantly predicted the happiness of Malaysian and Indonesian adults. Self-esteem partially mediated the association between religiosity, gratitude, and happiness. Multigroup analysis revealed cultural differences in the role of religiosity, gratitude, and self-esteem in the two countries. Among Indonesian adults, religiosity, gratitude, and self-esteem each showed a significant direct association with happiness. In contrast, in the Malaysian context, religiosity and gratitude contributed more strongly to self-esteem, which in turn predicted happiness.

**Conclusions:**

This study highlights both shared and culture-specific pathways to happiness in Malaysia and Indonesia. Findings emphasise the role of cultural context in shaping psychological mechanisms of happiness and suggest that interventions may be more effective when tailored to the distinct ways religiosity and gratitude operate across societies.

## Background

Happiness is a multidimensional construct encompassing both affective experiences, such as joy, contentment, pleasure, and cognitive evaluations, including life meaning and satisfaction [1–3]. In contemporary psychology, happiness is typically conceptualised under the broader construct of subjective well-being, which encompasses affective experiences, life satisfaction, and eudaimonic fulfilment [4, 5]. From this perspective, happiness goes beyond positive emotions. It encompasses engagement, relationships, meaning, accomplishments, and perseverance in the face of life’s challenges. These elements are captured in Seligman’s PERMA model, which positions happiness and well-being as central pillars of human flourishing, not merely the absence of distress but essential components of optimal functioning [6, 7]. Within this framework, gratitude fosters positive emotions and social connectedness, while religiosity contributes to a sense of meaning and a feeling of belonging.

From a psychological perspective, religiosity influences happiness not merely through belief endorsement or religious affiliation, but through the emotional and cognitive orientations it fosters. One disposition consistently identified as central to this process is gratitude. Religious teachings and practices, such as prayer, worship, and meaning making, encourage individuals to interpret life events as purposeful or divinely guided rather than random, thereby nurturing a generalized grateful disposition [8, 9]. Empirical evidence demonstrates that religiosity is positively associated with gratitude across cultures and faiths, including Indian multireligious samples, Iranian Muslims, Polish Christians, Vietnamese believers, and students in diverse settings [8, 10–12].

Gratitude fosters positive emotions, enhances life satisfaction, and buffers stress and negative affect, making it a key predictor of happiness [12–14]. While gratitude and religiosity exert direct effects on happiness, self-esteem may also mediate these relationships by reflecting relational and personal worth. According to Sociometer Theory, self-esteem serves as a psychological gauge of social acceptance and belonging, suggesting that higher levels of religiosity and gratitude may enhance happiness by boosting self-esteem [15]. Across cultures and methodologies, these findings converge on the view that religiosity and gratitude promote well-being, with self-esteem shaping the extent of this influence.

Together, these theoretical perspectives provide a foundation for examining self-esteem as a mediator of the relationships among religiosity, gratitude, and happiness across the two countries. Gratitude, religiosity, and self-esteem are key predictors of happiness, interacting in complex ways, with self-esteem mediating these effects. Previous research identifies these constructs as key predictors of happiness, often interacting in complex ways, with self-esteem serving as a mediator of well-being outcomes [16–18]. Building on this literature, the present study examines gratitude and religiosity as independent predictors of happiness, with self-esteem as the key mediator, to better understand culturally nuanced pathways to subjective well-being among Malaysian and Indonesian adults.

### Cross-cultural perspectives of happiness

Happiness is universally valued, but it is culturally shaped and influenced. In individualistic societies, it is tied to self-expression, achievement, and self-esteem, whereas in collectivist cultures it is linked to harmony, social connectedness, and emotional balance [19–21]. Global efforts to quantify happiness underscore its significance. For instance, the World Happiness Report has chosen happiness as one of the key indicators of social progress, even surpassing economic indicators in assessing human well-being [22]. Based on the ranking, Nordic countries consistently occupy the top ranks, with Finland, Denmark, Iceland, and Sweden leading in 2024. In comparison, Malaysia and Indonesia occupy middle positions, ranking 64th and 83rd, respectively, out of 147 countries. These numbers reflect not only material aspects but also social trust, community relationships, and perceptions of freedom. Moreover, it highlights the importance of understanding how cultural factors impact happiness in diverse contexts.

Although Malaysia and Indonesia share many similarities, such as collectivist orientations, multi-religious populations, and Islam as the majority faith, their cultural and social contexts diverge in ways that may shape happiness. Malaysia is characterised by marked ethnic and religious diversity, with significant Malay, Chinese, and Indian communities. This diversity is managed through institutionalised policies that embed Islam into legal and educational systems. As a result, religion is often politicised and plays a central role in shaping national identity [23–25]. In contrast, while Indonesia is the world’s largest Muslim-majority nation, it emphasises religious pluralism under the national philosophy of *Pancasila* and the motto *Bhinneka Tunggal Ika* (“Unity in Diversity”). The country is more ethnically homogenous, with national culture strongly influenced by the Javanese majority [26–28]. Governance is more decentralised, with religious life shaped by societal values and civil society organisations, rather than direct state control [29, 30]. These contrasts suggest that the psychological mechanisms linking religiosity, gratitude, self-esteem, and happiness may operate differently across the two societies, despite their shared collectivist and religious foundations.

### Religiosity and happiness

Empirical studies have consistently shown that religiosity is associated with higher happiness and well-being, primarily by providing meaning, community, and coping resources [31, 32]. Individuals who engage in religious practices such as prayer, forgiveness, or regular attendance at services tend to report higher levels of happiness and well-being [33, 34]. Both intrinsic religiosity and positive religious coping are strongly linked to greater happiness [35], while non-organised practices, such as personal devotion, offer similar benefits [36]. Conversely, spiritual struggles, including moral or interpersonal conflicts, are associated with reduced happiness [37]. However, this relationship varies depending on cultural context. While religiosity strongly predicts happiness in highly religious societies, evidence from more secular contexts suggests weaker or absent associations [38].

In Southeast Asia, religiosity plays a prominent role in daily life. In Indonesia, for instance, higher religiosity has been linked to greater life satisfaction, self-control, and happiness [39]. In Malaysia, religiosity similarly predicts quality of life, though the strength of the relationship varies across ethnic groups and life stages [40]. Despite these findings, comparative studies remain scarce. Both countries share Islamic traditions and collectivist values, but their political and religious structures differ significantly. Therefore, it is unclear whether religiosity contributes to happiness in similar or divergent ways across Malaysia and Indonesia.

### Gratitude and happiness

Gratitude, broadly defined as an emotional response to recognising the positive aspects of life, is strongly associated with happiness across cultures [41, 42]. It encourages individuals to focus on positive experiences and reinterpret events constructively, thereby reducing negative thoughts and enhancing resilience [41, 43]. Empirical studies have demonstrated that gratitude is positively associated with life satisfaction and is mediated by resources such as meaning in life, social support, and self-esteem [44]. Individuals high in gratitude also experience stronger connections between daily uplifts and positive emotions, leading to greater life satisfaction [45]. Experimental studies also confirm that gratitude interventions, such as journaling, can significantly boost happiness [44].

Cross-cultural evidence demonstrates that gratitude fosters positive emotions, resilience, and prosocial behaviour [42, 46], though its role varies across contexts. In Western samples, gratitude is often conceptualised as an individual trait, whereas in collectivist societies it is closely tied to social harmony and obligations [8]. In workplace contexts, gratitude also predicts happiness through psychological and social capital [47], suggesting its influence spans both personal and professional domains. Gratitude also plays a significant role in both Malaysia and Indonesia. In Malaysia, it predicts prosocial behaviours such as youth volunteerism and contributes to student happiness and well-being [48]. Among Indonesian adults, gratitude interacts with religiosity and self-compassion to shape subjective well-being [49]. These findings suggest that gratitude not only contributes directly to happiness but also functions in tandem with other cultural resources, such as religiosity and self-esteem.

Despite this evidence, little is known about how gratitude operates in Malaysia and Indonesia when examined simultaneously with religiosity and self-esteem. Comparative studies are particularly scarce, leaving open the question of whether gratitude’s role in happiness is universal across both societies and shaped by cultural context.

### Potential mediating role of self-esteem

Self-esteem, reflecting an individual’s evaluation of self-worth, is one of the most reliable predictors of happiness across cultures [50]. Higher self-esteem is associated with greater flexibility, social competence, and resilience, which in turn enhance well-being [51]. However, the strength of this association varies by culture. It tends to be stronger in individualistic societies, where self-worth is tied to personal achievement, compared to collectivist cultures, where self-esteem is often shaped by social harmony and group values [52]. In Malaysia, self-esteem is closely tied to social status, dignity, and community values, whereas in Indonesia, it is influenced by religiosity in predicting mental well-being and happiness [53, 54].

Research further suggests that, beyond its role as a direct predictor, self-esteem also functions as a mediator. Higher levels of self-esteem are associated with greater gratitude, which in turn enhances well-being and happiness [18]. Although the link between self-esteem and positive life outcomes is disputed [55, 56], evidence generally supports its importance for subjective happiness and life satisfaction. Individuals with higher self-esteem tend to report stronger relationships, greater emotional stability, and better coping mechanisms with stress [51].

Nevertheless, few studies have tested the mediating role of self-esteem in Southeast Asian contexts, where collectivist and religious traditions may shape self-worth differently [46, 57]. Examining this role in Malaysia and Indonesia will clarify whether self-esteem serves as a universal or culturally contingent mechanism linking religiosity and gratitude to happiness.

### Conceptual model and hypotheses

Despite increasing attention to well-being in Southeast Asia, few studies have systematically compared the psychological mechanisms of happiness in Malaysia and Indonesia. Given their shared collectivist traditions yet differing cultural and governance contexts, it remains unclear whether religiosity, gratitude, and self-esteem contribute to happiness in similar or distinct ways.

**Fig. 1:**
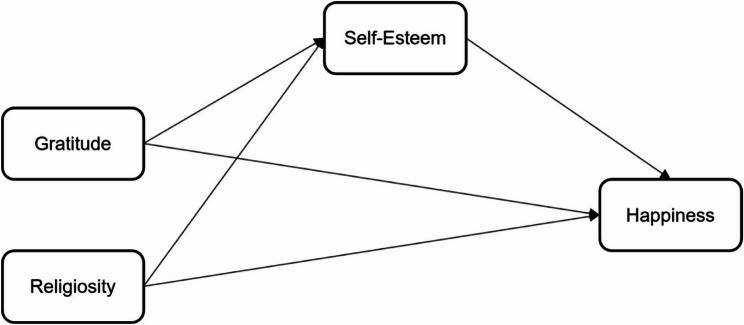
Hypothesised conceptual model

To address this gap, the present study tested a conceptual model examining the relationships between religiosity, gratitude, self-esteem, and happiness among Malaysian and Indonesian adults (Fig. 1). The model examined both direct associations and indirect pathways, with self-esteem hypothesised to mediate the effects of religiosity and gratitude on happiness. Additionally, cross-cultural comparisons were conducted to assess whether these structural relationships differ between Malaysian and Indonesian adults. Based on prior literature, the following hypotheses were proposed:

- Ha1:Religiosity is positively associated with happiness among Malaysian and Indonesian adults.
- Ha2:Gratitude is positively associated with happiness among Malaysian and Indonesian adults.
- Ha3:Self-esteem is positively associated with happiness among Malaysian and Indonesian adults.
- Ha4:Religiosity is positively associated with self-esteem among Malaysian and Indonesian adults.
- Ha5:Gratitude is positively associated with self-esteem among Malaysian and Indonesian adults.
- Ha6:Self-esteem positively mediates the association between religiosity and happiness among Malaysian and Indonesian adults.
- Ha7:Self-esteem positively mediates the association between gratitude and happiness among Malaysian and Indonesian adults.
- Ha8:The structural associations among religiosity, gratitude, self-esteem, and happiness differ across Malaysian and Indonesian adults.

## Methods

### Sample

The study sample was selected using a non-probability voluntary sampling technique. Voluntary sampling is an approach that relies on individuals who willingly contribute their thoughts and experiences to the research, a typical approach for online surveys. Only individuals interested in participating in the research and meeting the researchers’ inclusion criteria have been invited to participate in the survey. While practical for reaching large numbers of participants, voluntary sampling limits the representativeness of the findings, as the sample may not accurately reflect the broader Malaysian and Indonesian adult populations. Volunteers for this study were solicited online, via public posts, social media accounts, and communication applications such as WhatsApp and Telegram. At the end of the survey, 739 participants (300 Malaysians and 439 Indonesians) were included in this study. The inclusion criteria were as follows: (a) the respondents must be either Malaysian or Indonesian, and (b) the respondents must be 18 years or older. Researchers excluded responses with missing values or straight-lining issues, resulting in 730 valid responses for the analysis.

Overall, this study was conducted among 40.6% Malaysian and 59.4% Indonesian adults aged between 18 and 72 years. From these total numbers, 83.2% of the respondents belonged to the age category of young adults, aged below 40 years old, 15.9% were middle-aged adults, between 40 and 64 years old, and 1.0% were late adults, aged 65 years old and above. Approximately 23.7% of the respondents were male, and 76.3% were female. More than two-thirds of the respondents (74.9%) were single, 22.7% were married, 2.2% were divorced, and 0.2% were widows. In terms of education level, more than half of the respondents (52.3%) had a higher education (tertiary education), and the other 47.7% had a lower education (primary and secondary). Among young adults with a tertiary education level, 9.2% of the respondents had diplomas, 27.7% had bachelor’s degrees, 13.8% had master’s degrees, and 1.6% had a Doctor of Philosophy degree. The average household income for Malaysian adults was Malaysian Ringgit 9,779.73 (approximately USD 2,173.15), while that for Indonesian adults was Indonesian Rupiah 3,985,053.65 (approximately USD 246.11).

### Instrumentations

Data for this study were collected using a self-administered questionnaire. The questionnaire consisted of five sections, including demographic information such as gender, age, marital status, education level, and monthly household income. The main part of the survey included questions on happiness, gratitude, religiosity, and self-esteem. All instruments were administered in both English and Malay, or English and Indonesian versions, using a forward–backward translation process [58] to ensure linguistic and conceptual equivalence. The translations were pilot tested with 50 adults from both countries, with minor revisions made for clarity and accuracy.

Happiness was assessed using the Oxford Happiness Questionnaire Short Form [59]. The OHQ-SF is a unidimensional, 8-item, 6-point Likert scale, ranging from 1 (strongly disagree) to 6 (strongly agree). Total scores range from 8 to 48, with higher scores indicating greater happiness. Internal consistency was good across the total sample (α = 0.87), as well as for the Malaysian (α = 0.85) and Indonesian (α = 0.87) subsamples.

Gratitude was assessed with the Gratitude Questionnaire [60], a 6-item scale rated on a 7-point scale (1 = strongly disagree to 7 = strongly agree). Items 3 and 6 are reverse-coded. Higher total GQ-6 scores indicate higher levels of gratitude. The GQ-6 demonstrated good internal reliability for the total sample (α = 0.82), Malaysians (α = 0.87), and Indonesians (α = 0.87).

Religiosity was measured using the Centrality of Religiosity Scale [61]. This scale evaluates five dimensions of religiosity: intellectual (knowledge of religion and religiosity), ideological (belief in an otherworldly reality beyond everyday experiences), public practice (participation in social and religious rituals), private practice (engagement in solitary religious rituals), and religious experience (direct contact with an otherworldly reality). Each dimension was assessed using two items, resulting in a total of 10 items on the scale. Responses are given on a Likert scale, with higher scores reflecting stronger centrality of religiosity. Although the CRS-10 includes these five dimensions, previous studies have demonstrated that the subscales are highly intercorrelated and load onto a single higher-order factor representing the overall centrality of religiosity [61]. Therefore, consistent with the original validation and subsequent research [38, 46], the total CRS-10 score was used in the present study as an overall index of religiosity, ensuring consistency and comparability across the Malaysian and Indonesian samples. In this study, the CRS-10 showed excellent reliability for the total sample (α = 0.94), Malaysians (α = 0.90), and Indonesians (α = 0.84).

Self-esteem was assessed using the Rosenberg Self-Esteem Scale [62]. This 10-item scale is rated on a 4-point Likert scale (1 = strongly disagree to 4 = strongly agree), with both positively and negatively worded items. Total scores ranged from 10 to 40, with higher scores reflecting higher self-esteem. Internal consistency was acceptable for the total sample (α = 0.80), as well as for the Malaysian (α = 0.87) and Indonesian (α = 0.86) subsamples.

### Data collection procedure

A cross-sectional study was conducted to gather the data for this research. Before data collection, a pilot study was conducted with a total of 100 adults, consisting of 50 participants from Malaysia and 50 from Indonesia, to test the validity and reliability of the questionnaires. Based on the findings from the pilot study, several semantic refinements were made to enhance cultural appropriateness and clarity, including simplifying a few phrases and adjusting certain terms to ensure equivalent meaning across both languages. Syntactic adjustments were also implemented to improve sentence flow and readability. Following these modifications, the questionnaires underwent a second round of back-translation verification by bilingual experts to confirm conceptual and linguistic equivalence between the English, Malay, and Indonesian versions. Minor layout improvements were also introduced in the online questionnaire to enhance readability and user navigation. This process ensured that the instruments were clear, concise, and unambiguous. It helped identify and resolve potential issues so that the items were well understood and accurately measured the intended constructs [63]. Ethical approval was also obtained from the University Research Ethics Committee.

Participants were recruited through an online survey shared via Google Forms links on social media and communication platforms. All responses were collected anonymously to reduce potential social desirability bias. Prior to the main part of the questionnaire, respondents were informed of the study’s objectives, their roles, and the benefits of their participation. Online informed consent was obtained from all respondents, and no monetary incentives were provided. This selected sampling strategy was influenced by time, financial, and locality constraints, though these limitations did not affect the reliability of the statistical interpretations.

### Data analysis

Data analyses were conducted in several stages, from data exploration and normalisation, followed by descriptive statistics, Confirmatory Factor Analysis (CFA), and Structural Equation Modelling (SEM). First, data were screened to ensure completeness and accuracy. Responses with missing values or straight-lining were excluded. Normality was assessed using skewness and kurtosis values. Descriptive statistics were calculated for demographic variables.

Confirmatory Factor Analysis (CFA) was then performed to validate the measurement model, followed by Structural Equation Modelling (SEM) to test the hypothesised relationships. Model fit was evaluated using multiple indices, in line with established guidelines. Mediation effects were tested using bootstrapping procedures to estimate indirect effects of religiosity and gratitude on happiness through self-esteem. Bootstrapped indirect effects were estimated only for the hypothesised mediation pathways involving self-esteem; the indirect pathway from religiosity to happiness via gratitude was not specified in the present analytical model. For cross-cultural analysis, multigroup SEM was conducted to compare Malaysian and Indonesian samples. Measurement invariance was assessed sequentially prior to comparing structural paths across groups, ensuring that constructs were interpreted equivalently.

Participants in both countries were predominantly Muslim, reflecting the national religious composition of Malaysia and Indonesia, with only a small proportion representing other faiths. Given the limited representation of religious minorities, religious affiliation was not treated as a grouping variable in the analysis. Similarly, socioeconomic status (SES) was not included as a covariate, as the study focused on psychological constructs rather than demographic comparisons. These factors were considered in interpreting the findings and are acknowledged as potential sources of variability. All statistical analyses were performed using IBM SPSS Statistics Version 21 and AMOS Version 27.

## Results

### Assessment of data assumptions

The dataset was screened to ensure compliance with the statistical assumptions. All variables had skewness and kurtosis values below 2, except for religiosity, which was slightly higher but still below the acceptable threshold of less than 3 [64]. Therefore, the normality assumption was satisfied. Multivariate outliers were analysed using the Mahalanobis distance. A total of nine multivariate outliers were removed from the dataset, resulting in a final sample size of 730 valid cases for further analysis. Additionally, outlier screening using Cook’s Distance revealed no values exceeding the threshold of 1, confirming the absence of influential outliers. Multicollinearity was also examined, with Variance Inflation Factors (VIF) all below 10 and Tolerance values above 0.1, indicating no multicollinearity issues among the variables [65].

### Cross-national comparison of demographics

Table 1 shows the cross-national comparison of demographic profiles. Independent samples t-tests were conducted to examine gender differences in key variables within each national sample. For the Malaysian sample, a significant difference emerged for religiosity, t (289) = 2.63, *p* < 0.01, with male participants (M = 43.405, SD = 6.130) reporting higher religiosity than females (M = 40.820, SD = 7.672). No significant gender differences were found for other variables. For the Indonesian sample, significant gender differences were found for self-esteem, t (437) = 3.911, *p* < 0.001, with male participants (M = 29.647, SD = 4.728) reporting higher self-esteem than females (M = 27.244, SD = 5.552). A similar pattern was found for happiness, t (437) = 4.702, *p* < 0.001, where male participants (M = 37.828, SD = 5.849) scored higher than females (M = 34.574, SD = 6.121). The findings indicate that gender differences among Malaysians were limited to religiosity, whereas among Indonesians, significant gender differences were observed in self-esteem and happiness.

**Table 1:** Mean comparison among countries by sociodemographic characteristics

Variable	Religiosity		Gratitude		Self-esteem		Happiness	
	MY	ID	MY	ID	MY	ID	MY	ID
Gender								
Male	43.405 (6.130)	41.939 (6.765)	67.378 (7.446)	66.434 (8.292)	30.595 (5.938)	29.647 (4.728)	37.108 (6.298)	37.828 (5.849)
Female	40.820 (7.672)	42.018 (5.152)	66.013 (8.935)	65.671 (7.735)	30.115 (5.839)	27.244 (5.552)	35.871 (6.149)	34.574 (6.121)
*p*-value	0.004	0.902	0.239	0.396	0.544	0.000	0.139	0.000
Age								
< 40	41.038 (7.147)	41.538 (5.518)	65.180 (9.157)	65.147 (7.868)	28.545 (5.414)	27.275 (5.388)	35.137 (6.206)	34.629 (5.917)
40–64	42.685 (7.854)	46.256 (3.755)	69.288 (5.901)	72.256 (3.947)	34.685 (4.518)	32.488 (3.647)	38.767 (5.417)	41.558 (5.288)
≥ 65	42.143 (9.191)	-	71.429 (5.473)	-	34.857 (4.634)	-	40.857 (3.485)	-
*p*-value	0.253	0.000	0.001	0.000	0.000	0.000	0.000	0.000
Marital Status								
Single	40.653 (7.289)	41.328 (5.540)	64.989 (8.819)	64.736 (8.004)	28.663 (5.367)	26.695 (5.239)	35.156 (5.936)	34.141 (5.784)
Married	43.691 (7.239)	44.624 (4.825)	69.901 (6.715)	69.965 (5.549)	33.889 (4.952)	32.035 (4.185)	39.259 (5.130)	39.694 (5.724)
Divorced/ Widow	40.900 (7.370)	43.833 (4.792)	65.500 (10.330)	71.667 (5.888)	31.300 (8.538)	30.833 (4.070)	31.800 (9.976)	40.833 (6.555)
*p*-value	0.007	0.000	0.000	0.000	0.000	0.000	0.000	0.000

*MY* Malaysian, *ID* Indonesian

Concerning the age demographic, a one-way ANOVA was conducted to examine differences across age groups. For the Malaysian sample, the results showed no significant difference in religiosity among the three age groups. However, significant differences were found for gratitude, F (2, 288) = 7.800, *p* < 0.001. Post hoc comparisons using the Tukey HSD test showed that middle adults (participants aged between 40 and 64 years reported significantly higher levels of gratitude compared to those aged below 40 years. The same pattern was also found for self-esteem [F (2,288) = 38.293, *p* < 0.001] and happiness [F (2,288) = 54.241, *p* < 0.001], indicating that Malaysian older adults experienced greater self-esteem and happiness compared to younger age groups. For the Indonesian sample, independent samples t-tests were conducted since there were no participants aged above 65 years. The results revealed significant differences across all variables, with participants in the older age group (40–64 years) reporting higher levels of religiosity [t (437) = -5.468, *p* < 0.001], gratitude [t (437) = -5.842, *p* < 0.001], self-esteem [t (437) = -6.188, *p* < 0.001], and happiness [t (437) = -8.554, *p* < 0.001] compared to those aged below 40 years.

Across marital status, one-way ANOVA were conducted to examine group differences. For the Malaysian group, significant differences were found across marital status for all variables. Post hoc analyses revealed that single adults scored the lowest in religiosity [F (2, 287) = 5.052, *p* < 0.01], gratitude [F (2, 287) = 10.037, *p* < 0.001], and self-esteem [F (2, 287) = 27.322, *p* < 0.001], whereas for happiness, the divorced/widowed group reported the lowest scores [F (2, 287) = 16.808, *p* < 0.001]. For the Indonesian sample, the results also showed significant differences across all variables. Post hoc analyses indicated that single participants scored significantly lower in religiosity [F (2, 436) = 13.068, *p* < 0.001], gratitude [F (2, 436) = 18.093, *p* < 0.001], self-esteem [F (2, 436) = 39.439, *p* < 0.001], and happiness [F (2, 436) = 34.289, *p* < 0.001] compared to married and divorced/widowed participants.

In summary, the cross-national comparison revealed that gender, age and marital status influenced key variables differently across countries. Gender differences were found only in religiosity among Malaysians, whereas Indonesian males reported higher self-esteem and happiness. Older adults in both countries generally showed greater gratitude, self-esteem and happiness, while single adults consistently reported the lowest scores across most variables. Overall, demographic factors contributed to variations in psychological factors in both samples.

### Correlation analysis

Pearson’s correlation coefficients showed significant positive associations among all study variables. As shown in Table 2, all the study variables were significantly correlated, with correlation magnitudes ranging from 0.220 to 0.750. The positive correlations between all variables indicate that increases in religiosity, gratitude, and self-esteem are associated with higher levels of happiness.

**Table 2:** Correlations for all latent variables

Variable	1	2	3
1. Religiosity			
2. Gratitude	0.523***		
3. Self-esteem	0.220***	0.527***	
4. Happiness	0.338***	0.604***	0.750***

***p < 0.001; **p < 0.01; *p < 0.05

Pearson’s correlation tests revealed that religiosity (*r* = 0.338, *p* < 0.001), gratitude (*r* = 0.604, *p* < 0.001), and self-esteem (*r* = 0.750, *p* < 0.001) were positively and significantly correlated with happiness. Gratitude was most strongly correlated with self-esteem (*r* = 0.527, *p* < 0.001), while religiosity was the weakest correlated with self-esteem, yet still significant (*r* = 0.220, *p* < 0.001). Additionally, religiosity was also significantly related to gratitude (*r* = 0.523, *p* < 0.001). Thus, these findings provide initial support for Hypotheses 1, 2, 3, 4, and 5. These results reveal that Malaysian and Indonesian adults with higher levels of religiosity and gratitude tend to report greater happiness and stronger self-esteem. Furthermore, the findings suggest that Malaysian and Indonesian adults with stronger religiosity tend to cultivate greater gratitude in their lives, and that individuals with higher self-esteem are more likely to experience greater happiness.

### Measurement models

Confirmatory Factor Analyses (CFA) were conducted for the four latent constructs (religiosity, gratitude, self-esteem, and happiness). Model fit was evaluated using Chi-square and degree of freedom (χ²/df), RMSEA, CFI, and TLI. As shown in Table 3, all constructs demonstrated acceptable to marginally acceptable fit (χ²/df = 5.03–5.69, RMSEA ≤ 0.08, CFI ≥ 0.90, TLI ≥ 0.90). Although the χ²/df ratios slightly exceeded the preferred threshold of ≤ 5.00, the other fit indices met conventional criteria for adequacy, indicating that the constructs were conceptually coherent and unidimensional [66, 67].

**Table 3:** Fit indices for the study construct

Construct	χ^2^	df	χ^2^/df	CFI	TLI	RMSEA
Religiosity	170.203	30	5.673	0.961	0.942	0.080
Gratitude	206.109	41	5.027	0.949	0.932	0.074
Self-esteem	176.353	31	5.688	0.953	0.929	0.080
Happiness	89.127	17	5.243	0.941	0.903	0.076

*χ²* Chi Square, *df* degree of freedom, *CFI* Comparative Fit Index, *TLI* Tucker-Lewis Index, *RMSEA* Root Mean Square Error of Approximation

Following the establishment of unidimensionality and a good fit for each construct, the parcelling technique was applied to enhance the psychometric properties and simplify the subsequent structural equation model. For constructing with a unidimensional structure, item parcelling results in better-fitting solutions and less bias in estimates of structural [68] parameters [69]. The items were grouped based on their factor loading to ensure internal consistency within each parcel. Table 4 shows factor loadings and critical ratios (CR) for each parcel.

**Table 4:** Factor loadings and critical ratios of parcels

Construct	Parcel	Factor Loading	Standard Error (SE)	Critical Ratio (CR)	Significance (*p*-value)	Internal Consistency
Religiosity	Parcel 1	0.711				0.871
	Parcel 2	0.820	0.058	20.755	< 0.001	
	Parcel 3	0.859	0.060	21.614	< 0.001	
	Parcel 4	0.740	0.050	18.834	< 0.001	
	Parcel 5	0.801	0.060	21.323	< 0.001	
Gratitude	Parcel 1	0.796				0.874
	Parcel 2	0.801	0.029	22.817	< 0.001	
	Parcel 3	0.747	0.032	21.048	< 0.001	
	Parcel 4	0.748	0.029	21.063	< 0.001	
	Parcel 5	0.778	0.031	22.057	< 0.001	
Self-Esteem	Parcel 1	0.682				0.881
	Parcel 2	0.825	0.063	19.583	< 0.001	
	Parcel 3	0.810	0.059	19.288	< 0.001	
	Parcel 4	0.783	0.055	18.776	< 0.001	
	Parcel 5	0.774	0.063	18.583	< 0.001	
Happiness	Parcel 1	0.693				0.802
	Parcel 2	0.691	0.056	15.320	< 0.001	
	Parcel 3	0.735	0.069	15.946	< 0.001	
	Parcel 4	0.720	0.071	15.751	< 0.001	

***p < 0.001; **p < 0.01; *p < 0.05

All parcels demonstrate significant and strong standardised factor loadings (> 0.5) on their respective latent constructs. All critical ratios (CR) are greater than 1.96, proving that the observed parcel significantly contributes to the latent construct it is intended to measure. All obtained coefficient alphas exceeded 0.80, indicating internal consistency was high across all measures.

### Structural model

**Fig. 2:**
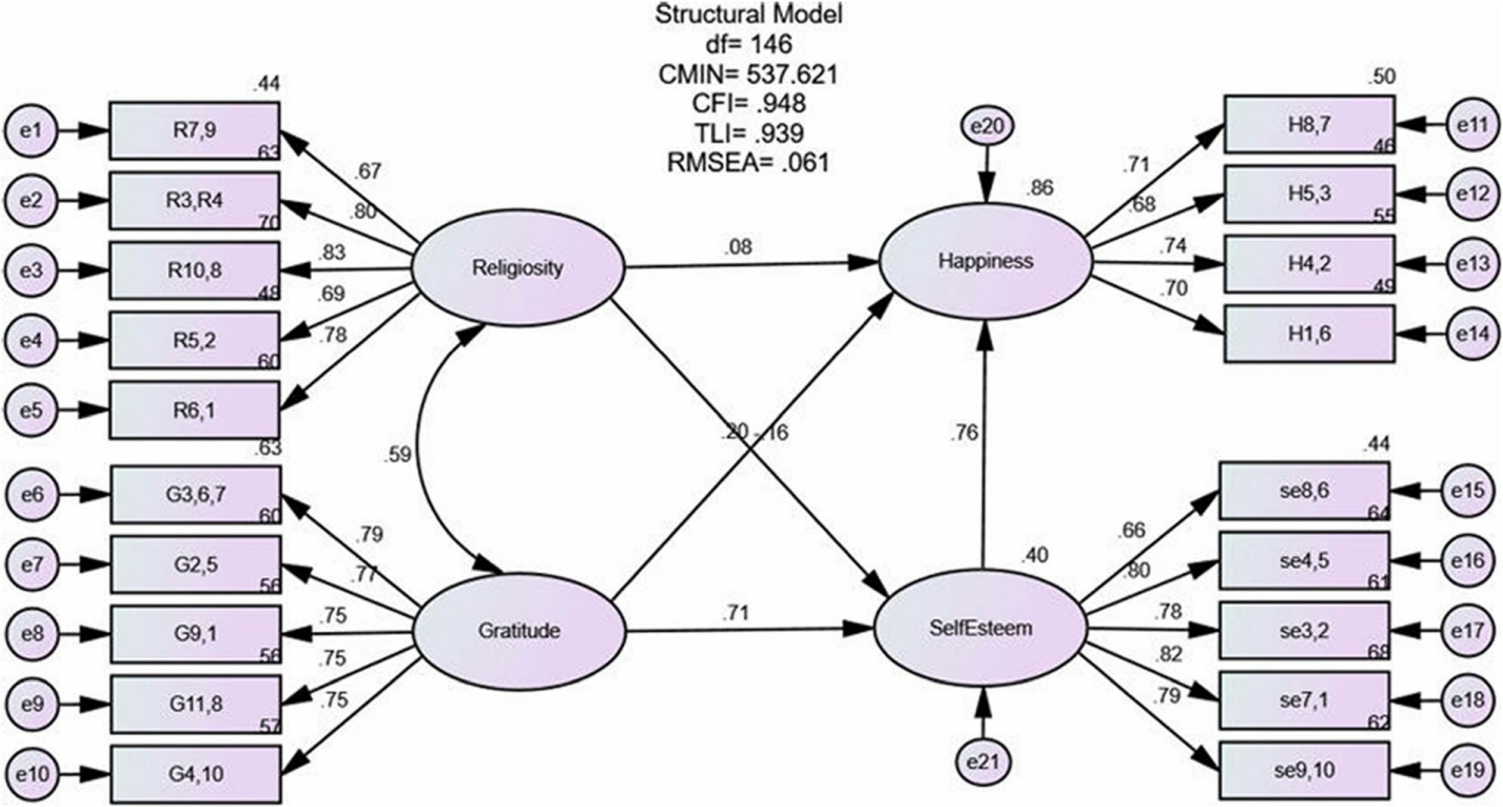
Structural model of religiosity and gratitude on happiness with self-esteem as mediator

All the parcelled models were then combined to form a full structural model to examine the relationship between the constructs. As shown in Fig. 2, the structural model showed a satisfactory fit (χ²=537.621, df = 146, χ²/df = 3.682, RMSEA = 0.061, CFI = 0.948, TLI = 0.939). The result of the SEM analysis for this structural model indicated that all the parameters were statistically significant at the 0.05 level and in the expected direction. Consequently, the model accounted for 40.0% of the variance in self-esteem and 86.0% of the variance in happiness, indicating strong explanatory power.

As shown in Table 5, religiosity (β = 0.081, *p* < 0.05), gratitude (β = 0.202, *p* < 0.001), and self-esteem (β = 0.760, *p* < 0.001) were all positively associated with happiness, indicating that individuals with higher levels of religiosity, gratitude, and self-esteem reported greater happiness. Gratitude was also positively associated with self-esteem (β = 0.712, *p* < 0.001). However, contrary to expectations, religiosity was negatively associated with self-esteem (β = -0.163, *p* < 0.001). This unexpected result suggests that, within this sample, higher religiosity may not directly enhance self-esteem and could even have an inverse relationship.

**Table 5:** Structural model path coefficients

Path	Estimate (β)	SE	CR	*p*
Religiosity → Happiness	0.081*	0.051	2.175	0.030
Gratitude → Happiness	0.202***	0.032	4.171	0.001
Self-esteem → Happiness	0.760***	0.083	13.556	0.001
Gratitude → Self-esteem	0.712***	0.026	11.941	0.001
Religiosity → Self-esteem	-0.163***	0.045	-3.376	0.001

*SE* standard error, *CR* critical ratio, *p* significance level

***p < 0.001; **p < 0.01; *p < 0.05

### Mediation analysis

Mediation analysis using bootstrapping further examines the significance of self-esteem as a potential mediator in the structural model. Bootstrapping was performed using 1,000 resamples, with a 95% confidence interval. Table 6 illustrates the structural model path coefficients, including direct, indirect, and total effects.

**Table 6:** Mediation analysis results

Effect type	Path	Standardized Estimate (β)	Standard Error (SE)	Bootstrap BC 95% CI			Interpretation
				Lower	Upper	Sig p	
Direct effect	R→H	0.111	0.051	0.010	0.155	0.027	significant
	G→H	0.132	0.032	0.104	0.299	0.003	significant
Indirect effect	R→SE→H	-0.124	0.040	-0.203	-0.047	0.002	Significant
	G→SE→H	0.541	0.042	0.466	0.632	0.001	significant
Total effect	R→H	-0.043	0.053	-0.143	0.060	0.445	Not significant
	G→H	0.743	0.044	0.657	0.826	0.002	Significant

*R* religiosity, *H* happiness, *SE* self-esteem, *G* gratitude

For religiosity, both the direct effect on happiness (CI: 0.010, 0.155) and the indirect effect through self-esteem (CI: -0.203, -0.047) were statistically significant, as their confidence intervals did not include zero. This finding suggests that self-esteem partially mediates the relationship between religiosity and happiness. Thus, hypothesis 6 was partially supported but in an unexpected direction. However, the total effect of religiosity on happiness was nonsignificant (CI: − 0.143, 0.060), suggesting that the negative indirect pathway via self-esteem offset the small positive direct effect of religiosity on happiness.

Regarding gratitude, both the direct effect (CI: 0.104, 0.299), the indirect effect via self-esteem (CI: 0.466, 0.632), and the total effect (CI: 0.657, 0.826) were statistically significant. This result suggests that self-esteem partially mediates the relationship between gratitude and happiness. Thus, hypothesis 7 was also supported. In other words, gratitude not only directly enhanced happiness but also contributed indirectly through its positive influence on self-esteem.

### Multigroup analysis

Prior to cross-cultural comparisons, measurement invariance was tested sequentially, confirming that the constructs were measured equivalently across Malaysian and Indonesian adults. As shown in Table 7, the unconstrained model demonstrated good fit (χ²=719.830, df = 292, RMSEA = 0.045, TLI = 0.935, CFI = 0.944), and the constrained model also showed adequate fit (χ²=733.670, df = 297, RMSEA = 0.045, TLI = 0.935, CFI = 0.943). A significant chi-square difference (∆χ² = 13.840, ∆df = 5, *p* = 0.017) indicated that the structural paths differ across groups, suggesting cultural differences in the relationships among the constructs. Thus, Hypothesis 8 was supported.

**Table 7:** Multigroup analysis across nationality

Moderator		χ^2^	df	CV	∆χ^2^
Nationality	Unconstrained	719.830	292	11.070	13.840**
	Constrained	733.670	297		

*χ²* Chi Square, *df* degree of freedom, *CV* Critical Value

As shown in Table 8, follow-up comparisons revealed that the overall effects of religiosity (∆χ²=1.480, *p* > 0.05) and gratitude (∆χ²=1.210, *p* > 0.05) on happiness did not significantly differ between Malaysian and Indonesian samples. In contrast, significant group differences emerged for the paths from self-esteem to happiness (Δχ² = 8.760, *p* < 0.01), religiosity to self-esteem (Δχ² = 4.680, *p* < 0.05), and gratitude to self-esteem (Δχ² = 4.040, *p* < 0.05), indicating that these relationships vary across the two cultural contexts.

**Table 8:** Path coefficients in the structural model across nationality

Path	Standardised Regression Weight		∆χ^2^	*p*-value
	Malaysian	Indonesian		
Religiosity → Happiness	0.039	0.094**	1.480	0.224
Gratitude → Happiness	0.297***	0.136*	1.210	0.271
Self-esteem → Happiness	0.659***	0.823***	8.760**	0.003
Religiosity → Self-esteem	-0.268***	-0.020	4.680*	0.031
Gratitude → Self-esteem	0.812***	0.605***	4.040*	0.045

SE = ***p < 0.001; **p < 0.01; *p < 0.05

Results also showed that specific paths varied across groups. Religiosity predicted happiness only in the Indonesian group (β = 0.094, *p* < 0.01), but not in the Malaysian group (β = 0.039, *p* > 0.05). Similarly, religiosity significantly predicted self-esteem only in the Malaysian group (β = − 0.268, *p* < 0.001), but was not significant in the Indonesian group (β = − 0.020, *p* > 0.05). Overall, for the Indonesian group, both religiosity (β = 0.094, *p* < 0.01) and self-esteem (β = 0.823, *p* < 0.001) were stronger predictors of happiness compared to the Malaysian group. In contrast, for Malaysians, gratitude contributed more strongly to both happiness (β = 0.297, *p* < 0.001) and self-esteem (β = 0.812, *p* < 0.001), highlighting cultural differences in the sources of happiness between the two groups.

## Discussion

This study confirmed that religiosity, gratitude, and self-esteem are significant predictors of happiness. By examining these factors simultaneously, the findings underscore happiness as a multidimensional outcome influenced directly and indirectly by individual beliefs, personal traits, and self-perceptions. This interpretation aligns with positive psychology perspectives, which emphasise the integration of personal and cultural resources in sustaining life satisfaction.

Across both countries, religiosity was associated with happiness, consistent with prior research showing that religious belief and practice provide meaning in life, transcendence, and stronger social connectedness [32–34]. However, this contribution was relatively modest compared to other factors, suggesting that religiosity plays a supportive rather than central role in explaining happiness. Gratitude, in contrast, emerged as a stronger and more consistent predictor of happiness, echoing past studies [45, 69]. Gratitude is a powerful emotional trait that encourages adults to focus on the positive aspects of life. Accordingly, adults who cultivate gratitude tend to report greater happiness and stronger relational connectedness. Self-esteem was another robust predictor of happiness, reinforcing previous evidence that individuals with higher self-esteem are better equipped to cope with life challenges, build supportive networks, and sustain psychological resilience [50].

The present findings should be interpreted in light of established theoretical and empirical models that conceptualize gratitude as a central psychological mechanism linking religiosity to happiness and broader well-being. Although religiosity demonstrated a direct association with happiness in the current study, substantial prior research suggests that this relationship is largely explained by the emotional and cognitive orientations fostered through religious engagement. Religious beliefs and practices commonly emphasize thankfulness, recognition of blessings, and the interpretation of life events as meaningful or divinely guided, thereby cultivating a generalized disposition toward gratitude [8, 9]. This grateful disposition, in turn, enhances happiness by broadening positive affect, strengthening psychological resilience, and promoting adaptive emotional regulation and life satisfaction [70].

Empirical evidence across diverse cultural and contextual settings consistently supports gratitude as a mediator between religiosity and well-being. For example, gratitude has been shown to mediate the association between religiosity and life satisfaction and reduced depressive symptoms among U.S. undergraduates and adults formerly raised by grandparents [12], psychological well-being among Chinese college students in Hong Kong [11], and self-esteem and life satisfaction among Latter-day Saint Polynesian Americans [13]. Recent studies further demonstrate this mechanism in occupational and clinical contexts, including Polish workers and members of addiction recovery groups, where spirituality or religious practices predict well-being indirectly through gratitude [14, 71]. Across these populations, converging evidence indicates that religiosity or spirituality enhances happiness and well-being largely by fostering gratitude, sometimes alongside related constructs such as hope or forgiveness. In line with this literature, the current findings align with contemporary models by highlighting gratitude as a core psychological construct closely intertwined with religiosity and happiness across Malaysian and Indonesian samples. Although gratitude was not formally tested as a mediator in the present analyses, its robust theoretical grounding and consistent empirical support in prior research provide a compelling explanatory framework for understanding the observed association between religiosity and happiness.

Beyond being a direct predictor, self-esteem also served as a partial mediator between religiosity and happiness, as well as between gratitude and happiness. This dual role underscores self-esteem as both a direct enhancer of well-being and an explanatory mechanism linking cultural values to happiness. This finding is consistent with Sociometer Theory [8], which conceptualises self-esteem as a gauge of social belonging and relational value, suggesting that religiosity and gratitude may enhance happiness partly by reinforcing feelings of acceptance and self-worth. However, a multi-group analysis indicated that the mediating effect of religiosity on self-esteem was significant only among Malaysian participants, suggesting that the psychological role of religiosity may differ across cultural contexts. This interpretation also aligns with the PERMA model [72], particularly the ‘Meaning’ component. While religiosity is not an explicit element of the PERMA model, it can contribute to well-being by fostering a sense of meaning and purpose in life. Gratitude supports the ‘Positive Emotion’ component by nurturing optimism and contentment, whereas self-esteem reflects ‘Accomplishment’ and relational confidence.

A substantial body of research supports the idea that religiosity contributes to well-being by fostering meaning and purpose in life, aligning closely with the ‘Meaning’ pillar of Seligman’s PERMA model. Religiosity is repeatedly shown to be positively associated with a sense of meaning, purpose, and coherence, which in turn enhances psychological well-being and happiness [73, 74]. The partial mediation suggests that religiosity and gratitude influence happiness both directly and indirectly by boosting self-esteem. However, additional mechanisms, such as meaning in life, social support, or resilience, likely operate alongside self-esteem [75]. Importantly, the strength of the link between self-esteem and happiness may vary across cultures. In highly religious contexts, the role of self-esteem may be somewhat weaker, as well-being is also sustained by spiritual or communal mechanisms [76]. These complexities underscore the need for future longitudinal and mixed-method research to explore alternative mediating processes and to capture the dynamic interplay between cultural resources and happiness.

The cross-cultural comparison further clarified these dynamics. Among Indonesians, religiosity was modestly but directly linked to happiness, suggesting that religious practices and commitments such as observance, recitation, and prayers provide an immediate sense of satisfaction and well-being [77]. Among Malaysians, by contrast, religiosity contributed more indirectly, with both religiosity and gratitude strengthening self-esteem, which then enhanced happiness [78]. This pattern suggests that while both groups derive psychological benefits from religiosity, Indonesians gain more direct emotional satisfaction. In contrast, Malaysians tend to internalise religiosity and gratitude in ways that first shape their self-worth before influencing their happiness.

Although no significant group differences were observed in the direct effects of religiosity and gratitude on happiness, the effect of self-esteem on happiness was stronger among Indonesians. One explanation may lie in socioeconomic conditions. Indonesia has a relatively lower average income and social mobility compared to Malaysia [79], and this factor may heighten reliance on internal psychological resources such as self-esteem as key determinants of well-being. This interpretation aligns with Self-Determination Theory [80], which emphasises competence and value as central drivers of flourishing. Importantly, self-esteem was a robust predictor of happiness in both groups, suggesting that interventions designed to strengthen self-esteem could have broad benefits for adults in both contexts.

Significant group differences also emerged in the paths from religiosity and gratitude to self-esteem. This study found that gratitude had a strong and positive effect on self-esteem in both samples, with a more pronounced impact among Malaysians. This finding aligns with a growing body of research showing that gratitude is closely linked to higher self-worth and positive self-views [44, 81]. Gratitude encourages individuals to recognise and appreciate the kindness of others and the positive aspects of their lives, which can foster a sense of being valued and supported. This process enhances self-esteem by affirming individuals’ sense of worthiness to receive care, kindness, and good outcomes [82]. The stronger effect of gratitude on self-esteem among Malaysian adults may reflect cultural values that emphasise social harmony and mutual obligation, making gratitude a potent mechanism for reinforcing positive self-concept. Self-esteem among Malaysians may be more sensitive to religious commitments and gratitude practices [83]. Moreover, gratitude has been shown to promote prosocial behaviours, such as volunteerism and charitable giving (e.g., *‘sadaqah’*), which in turn foster self-worth and happiness in the collectivistic contexts like Malaysia [84]. For Indonesians, by contrast, religiosity exerted a more direct effect on happiness, with self-esteem playing a less central mediating role [57, 85]. Despite these differences, self-esteem consistently emerged as a universal predictor of happiness across both groups, underscoring its fundamental role in psychological well-being.

One of the most unexpected findings was the negative association between religiosity and self-esteem. Although religiosity and self-esteem were positively correlated at the bivariate level, the negative path coefficient in the Structural Equation Modelling indicates a suppression effect, where the inclusion of gratitude and happiness alters the direction of the relationship. Although present in both groups, this effect was particularly salient among Malaysians. This finding is counterintuitive, as religiosity is often expected to enhance happiness and well-being. One possible explanation may lie in the multidimensional nature of religiosity. When controlling for happiness and gratitude, higher religiosity possibly heightens moral self-awareness, leading adults to be more self-critical or to perceive themselves as falling short of religious expectations, which in turn lowers their self-evaluation [48, 76]. Although the Centrality of Religiosity Scale (CRS-10) used in the present study does not directly assess moral self-awareness, this interpretation is supported by research suggesting that religious commitment can sometimes evoke guilt or feelings of inadequacy when individuals struggle to meet perceived spiritual obligations [86].

The demographic composition of the current sample, which younger, single female participants dominate, may also partly explain this pattern. For younger adults, religiosity may function more as an external social expectation or moral standard than an internalised source of self-worth, resulting in weaker or even negative associations with self-esteem [87]. In collectivist contexts, religiosity may also emphasise modesty and humility, reflected in the concept of *tawadhuʿ* (humility), which may lead individuals to report lower explicit self-esteem yet maintain similar levels of happiness and well-being [55, 88, 89]. However, gratitude appeared to offset this effect by enhancing self-esteem, suggesting that relational and social recognition can buffer against the humility-related constraints of religiosity. These findings suggest that interventions targeting self-esteem in highly religious populations may benefit from reframing self-esteem as compatible with religious humility, emphasising inner dignity, personal growth, and self-compassion rather than pride or superiority.

Overall, the cross-cultural comparison revealed that religiosity and gratitude played a more prominent role in directly influencing happiness among Indonesians. In contrast, in Malaysia, religiosity and gratitude exerted a stronger influence on self-esteem, which subsequently contributed to happiness. This difference may reflect variations in cultural and religious expression, where Indonesians may derive a more immediate sense of life satisfaction from their religious commitment. At the same time, Malaysians may internalise religiosity and gratitude in ways that shape their self-concept before impacting their happiness. These findings highlight the importance of considering cultural context when designing interventions to promote well-being and happiness. For Indonesians, programs that strengthen religious engagement may directly enhance happiness, whereas for Malaysians, initiatives that link religious and gratitude practices to personal growth and self-acceptance may be more effective in fostering long-term happiness.

Importantly, the present study explicitly recognises gratitude as a theoretically grounded explanatory mechanism underlying the relationship between religiosity and happiness. Prior research has consistently demonstrated that gratitude mediates this association by translating religious beliefs and practices into adaptive emotional responses that enhance well-being. In the context of the present findings, gratitude is therefore interpreted as an explanatory process supported by extensive prior mediation research, rather than as an empirically tested mediating pathway in the current analytical model. By situating gratitude within this mechanistic framework, the study contributes to the growing body of literature that emphasises emotional pathways rather than belief systems alone as central to understanding how religiosity promotes happiness. This clarification strengthens the theoretical coherence of the study and aligns it with contemporary psychological models of religiosity and well-being.

## Limitations

This study presents important findings, but they should be interpreted considering several limitations. The reliance on non-probability voluntary sampling may restrict generalizability, as participants were limited to those willing to complete an online survey, potentially excluding groups with limited internet access or lower motivation to participate.

Moreover, the demographic composition of the sample was uneven, with a high concentration of younger, single female participants, which may have influenced the results and limited the representativeness of the findings across broader age, gender, and marital groups. The study did not control for religious denomination or socioeconomic background, as most participants identified as Muslim and reported relatively similar SES levels. These contextual factors may influence religiosity and self-esteem and should be examined in future research.

Furthermore, the exclusive use of self-report measures introduces potential response biases, particularly in socially sensitive domains such as religiosity and happiness. The study also did not include other psychosocial factors such as personality traits, meaning in life, optimism, resilience, or cultural values, which may further explain individual differences in happiness. Although gratitude is theoretically and empirically supported as a key psychological mechanism linking religiosity and happiness, it was not formally tested as a mediator in the present analytical model. This limits causal inferences regarding the indirect pathway through gratitude and highlights an important direction for future research.

Future studies should consider probability-based or mixed sampling methods to achieve greater demographic diversity, include additional psychosocial variables, and complement self-report data with behavioural or qualitative approaches. It would also be valuable to explore which specific dimensions of religiosity most strongly influence well-being and to employ longitudinal or mixed method designs to better capture the dynamic relationships among religiosity, gratitude, self-esteem, and happiness. Future longitudinal or experimental studies could explicitly model the pathway from religiosity to happiness through gratitude to examine whether this well-established mechanism operates similarly across Malaysian and Indonesian cultural contexts.

## Conclusion

Taken together, the findings in this study highlight the cultural nuances in how religiosity and gratitude shape happiness across the two countries. In Indonesia, religiosity showed a modest but significant association with happiness, whereas in Malaysia, its influence was more indirect, operating primarily through self-esteem. Gratitude also played a stronger role in enhancing self-esteem among Malaysians. These findings underscore the importance of considering cultural and contextual factors when examining the psychological mechanisms underlying happiness, as the same predictors may operate through different pathways or vary in strength across populations.

Theoretically, the results contribute to a more nuanced understanding of how religiosity and gratitude are embedded within cultural contexts. They also refine Sociometer Theory and extend the PERMA framework by demonstrating that the mediating role of self-esteem in happiness is shaped by cultural self-construal, highlighting how relational and spiritual values influence happiness in collectivist societies. Practically, results suggest that interventions aimed at promoting happiness may be more effective when tailored to reflect the distinctive ways these factors function across societies, by emphasising religious engagement in Indonesia and strengthening gratitude-based self-esteem programs in Malaysia. Tailoring interventions to reflect the diverse ways in which religiosity and gratitude function across different contexts can enhance their relevance and effectiveness.

## Data Availability

The datasets used and analysed during this study are available from the corresponding author upon request.
